# Study on the effect of ascorbic acid on the biosynthesis of pigment and citrinin in red yeast rice based on comparative transcriptomics

**DOI:** 10.3389/fmicb.2024.1460690

**Published:** 2024-09-10

**Authors:** Zhilin Jiang, Yunxun Duan, Qianqian Yin, Jing Zhang, Jing Chen, Jingsha Lan, Chen Xiao, Xian Tang, Xinhui Wang, Yong Zuo

**Affiliations:** ^1^Key Laboratory of the Evaluation and Monitoring of Southwest Land Resources (Ministry of Education), Sichuan Normal University, Chengdu, Sichuan, China; ^2^College of Life Science, Sichuan Normal University, Chengdu, China; ^3^College of Food and Biological Engineering, Chengdu University, Chengdu, China; ^4^Chengdu National Agricultural Science and Technology Center, Chengdu, China

**Keywords:** red yeast rice, pigment, citrinin, comparative transcriptomic, biosynthesis

## Abstract

Pigment is one of the most important metabolites in red yeast rice. However, citrinin may accumulate and cause quality security issues. In the present study, the effect of ascorbic acid (EAA) on the pigment and citrinin was studied, and the metabolic mechanism was discussed using comparative transcriptomics. The introduction of EAA increased the pigment by 58.2% and decreased citrinin by 65.4%. The acid protease activity, DPPH scavenging rate, and total reducing ability also increased by 18.7, 9.0, and 26.7%, respectively. Additionally, a total of 791 differentially expressed genes were identified, and 79 metabolic pathways were annotated, among which carbon metabolism, amino acid metabolism, and fatty acid metabolism were closely related to the biosynthesis of pigment and citrinin. Ethanol dehydrogenase (*M pigC*), oxidoreductase (*M pigE*), reductase (*M pigH*), and monooxygenase (*M pigN*) may be related to the increase of pigment. *ctnC* and *pksCT* contributed to the decline of citrinin.

## 1 Introduction

Red yeast rice, fermented with *Monascus* using rice as the substrate, is a dual-use product for medicine and food (Xun et al., 2024). *Monascus* pigment, a natural pigment extracted from fermented red yeast rice, offers high nutritive value and can serve as a replacement for nitrite, amaranth red, and other potentially carcinogenic and teratogenic pigments, having widely applied in vinegar brewing, food preservation, and as a food additive (Zhang et al., 2024). *Monascus* pigment also exhibits a variety of biological activities, such as antibacterial, antitumor, and antioxidant properties. These qualities make it a valuable option for applications in cosmetics, pharmaceuticals, and other extended fields (Shi et al., 2022; Yang et al., 2024). However, during the fermentation of red yeast rice, citrinin inevitably accumulates to different degrees. Citrinin, the molecular formula shown in Supplementary Figure S1, is a kind of fungal toxin belonging to the class of polyketide compounds, mainly produced by three genera: *Penicillium*, *Aspergillus,* and *Monascus* (de Oliveira Filho et al., 2017; Qin et al., 2023). Citrinin is nephrotoxic and hepatotoxic to mammals and can cause deformities, cancers, and mutations (Liu H Q, et al., 2021). In March 2024, the red yeast rice poisoning incident in Japan may be related to citrinin. Therefore, citrinin control during the fermentation of red yeast rice has become a hot topic. It is essential to regulate the secondary metabolites of *Monascus* to obtain high-quality products.

The U.S. Food and Drug Administration (FDA) and Japan have stipulated that the maximum limit of citrinin in *Monascus*-fermented products is 2 mg/kg. In China, citrinin content cannot exceed 0.05 mg/kg (National Development and Reform Commission of the People’s Republic of China, 2007). Currently, research on improving *Monascus* pigment and reducing citrinin mainly depends on strains (Zhang et al., 2024), fermentation regulation (Zhou et al., 2020; Wang et al., 2023), and molecular-level regulation (Liu et al., 2020). However, their application in industrial production needs further research and verification.

To further promote pigment production and control the content of citrinin, a new strategy has emerged, which involves regulating fermentation by exogenously adding regulatory factors (Huang Z, et al., 2023). Ascorbic acid (AA) is one of the major water-soluble antioxidants naturally existing in food, playing an important role in reducing cellular oxidative stress caused by the imbalance between reactive oxygen species (ROS) and antioxidant defense (Konings et al., 2024). EAA can also scavenge ROS and inhibit lipid peroxidation, which in turn protects the normal cellular metabolism from oxidative damage caused by ROS and peroxides (Pehlivan, 2017; Das et al., 2023; Moradi et al., 2024). Wei reported that the addition of EAA decreased the citrinin by 67.6% and increased the pigment by 29.3%. Both intracellular and extracellular antioxidant activities exhibited good ability, and the ratio of unsaturated and saturated fatty acids increased (Wei et al., 2022). EAA may activate the CREB cycle and induce a decrease of citrinin precursors produced by malonyl-CoA, further inhibiting the synthesis of citrinin. However, the molecular mechanism of EAA toward red yeast rice fermentation remains unclear.

**Figure 1:**
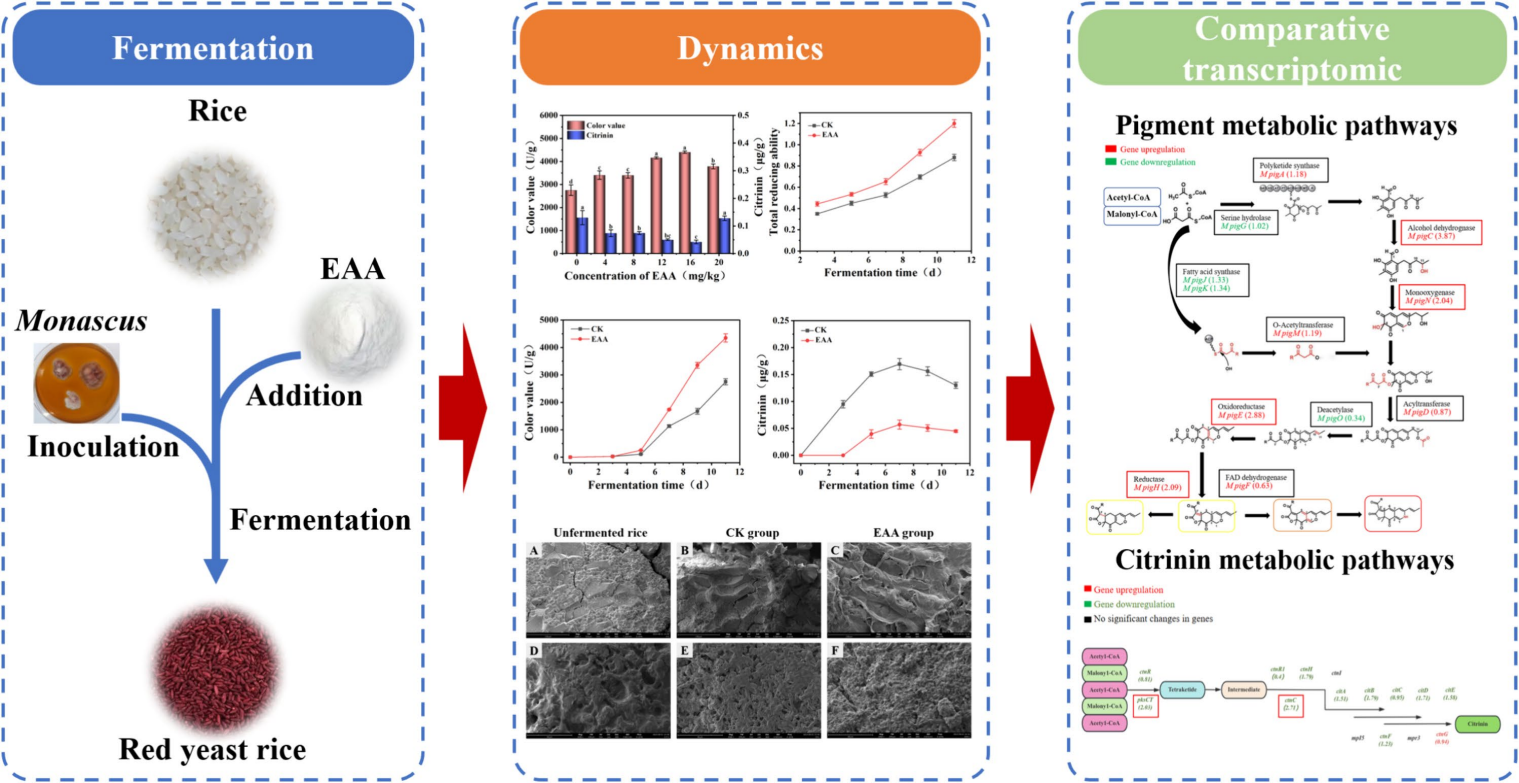
Scheme of the effect of EAA on the fermentation of red yeast rice.

In the present study, the effect of EAA on the *Monascus* pigment and citrinin during the solid-state fermentation of red yeast rice was conducted (Figure 1). The dynamic changes in the physical and chemical indices of red yeast rice under the optimal EAA concentration were analyzed. Additionally, comparative transcriptomics was applied to preliminary analyze the molecular mechanism of EAA regulation on *Monascus* pigment and citrinin.

## 2 Materials and methods

### 2.1 Strain, medium, and culture conditions

Strain: *Monascus purpureus*, named M7-5, was screened from fermented rice. Seed medium: 13.1 g of malt infusion powder was introduced into 100 mL of water and sterilized at 121°C for 15 min. Solid fermentation medium: 20 g of washed and soaked rice was dispensed into 250 mL conical flasks and sterilized at 121°C for 15 min.

A measure of 10% of M7-5 seed medium with a concentration of 1 × 10^6^ CFU/mL was added into the solid fermentation medium, which was then put into a mold incubator for cultivation at 30°C for the first 3 days and 28°C for the last 8 days. At the end of the fermentation, the red yeast rice was dried at 55°C.

### 2.2 Color value and citrinin

The color value was detected by referring to GB1886.19–2015, which is the standard for food additives, specifically red yeast rice (National Health Commission of the People’s Republic of China, 2015).

Citrinin was determined by referring to the method of C18 solid-phase extraction column purification-high performance liquid chromatography of GB 5009.222–2016 (National Health Commission of the People’s Republic of China, 2016).

Sample extraction: First, 1 g of sample was added into 5 mL of methanol–water solution (70∶30). After shaking for 30 min, the solution was centrifuged at 8000 r/min for 5 min. The residue was then added to 5 mL of methanol–water solution (70∶30) and extracted by shaking for 30 min. After centrifuging at 8000 r/min for 5 min, the supernatant was combined. Then, the supernatant was diluted to 15 mL with purified water, agitated for 1 min, and allowed to stand for 2 min. The solution was filtered using the microfiber membrane filter paper and concentrated to 1 mL at 50°C under vacuum conditions. Finally, the sample was filtered using a 0.22-μm microporous organic filter membrane for HPLC analysis.

Chromatographic conditions: The chromatographic separation was performed on an Agilent ZORBAX-SB-aq column (4.6 × 100 mm, 2.6 μm). A fluorescence detector was applied with detection wavelengths of λex = 331 nm and λem = 500 nm. The column temperature was 28°*C. mobile* phases A and B were phosphoric acid aqueous (pH 2.5) and acetonitrile, and the ratio of the mobile phases A and B was 33:67. The flow rate was 1 mL/min. The injection volume was 20 μL.

### 2.3 Antioxidant activity

A measure of 0.05 g of the sample was added into 10 mL of ethanol solution (70%) and placed in a water bath at 60°C for 1 h. After filtration and centrifugation (5,000 r/min, 10 min), the sample extract was obtained.

For the DPPH radical scavenging ability, 1 mL of the 10-fold diluted sample was mixed with 3 mL of DPPH–ethanol solution (0.1 mmol/L). The solution was reacted at room temperature in the dark condition for 30 min. The absorbance of the solution was then measured at 517 nm, using a 70% ethanol solution as the blank group.

For hydroxyl radical scavenging ability, 1 mL of the 10-fold diluted sample was mixed with 1 mL of FeSO_4_ (2 mmol/L), 1 mL of ethanol–salicylic acid (9 mmol/L), and 1 mL of H_2_O_2_ (8.8 mmol/L). The solution was reacted for 30 min at 37°C. The absorbance value of the solution at 510 nm was determined using a 70% ethanol solution as the blank group.

For total reducing power, 1 mL of the 10-fold diluted sample was mixed with 2.5 mL of phosphate buffer (0.2 mol/L, pH 6.6) and 2.5 mL of potassium ferricyanide (1%). The solution was placed in a water bath at 50°C for 20 min. Then, 1% trichloroacetic acid was added and the solution was centrifuged at 3000 r/min for 10 min. Mix the 2.5 mL supernatant, 2.5 mL distilled water and 0.5 mL FeC1_3_ (0.1%), and the absorbance value of the solution at 700 nm was determined using a 70% ethanol solution as the blank group.

### 2.4 Functional enzyme activities

The determination of superoxide dismutase (SOD) enzyme activity was determined by referring to National Health Commission of the People’s Republic of China (2003). The iodine-amylase colorimetric assay kit purchased from Nanjing Jiancheng Bioengineering Institute was used for the determination of α-amylase activity. The protease activity was determined by referring to GB/T23527-2009 (State Administration for Market Regulation of the People’s Republic of China, 2023).

### 2.5 Transcriptomic sequencing

The red yeast rice, with and without the addition of EAA, fermented for 36 h was collected for RNA extraction. The total RNA was extracted using TRIzol® reagent according to the manufacturer’s instructions (Invitrogen, United States). RNA concentration was assessed using Nanodrop®2000. RNA quality was tested using the Agilent 2,100 Bioanalyzer. The high-quality RNA samples (OD_260/280_ = 1.8 ~ 2.0, OD_260/230_ ≥ 2.0) were selected for the construction of the sequencing library. The sequencing of the library was performed on NovaSeq 6,000 (Illumina, United States) sequencing platform. The differentially expressed genes between the two groups were screened according to the criteria of a *p*-value of <0.05 and |log_2_FoldChange| > 0. The differentially expressed genes were further compared to the Gene Ontology (GO)^1^ and Kyoto Encyclopedia of Genes and Genomes (KEGG)^2^ databases to analyze the biological functions and pathways. The comparative transcriptomic analysis was performed to reveal the key genes related to the synthesis of pigment and citrinin.

### 2.6 RT-qPCR validation of key genes

The total RNA from *Monascus purpureus* was extracted using TRIzol reagent (TaKaRa MiniBEST, Beijing, China). The reverse transcription of RNA was conducted using the PrimeScript™ RT reagent kit with gDNA Eraser Kit. The gene expression was determined by RT-qPCR using the SYBR Premix Ex Taq II (TaKaRa) and ABI StepOne thermocycler (Applied Biosystems, NY, United States). GAPDH was applied as the reference gene, and the primer sequences are listed in Supplementary Table S1.

### 2.7 Statistical analysis

All experiments were repeated three times, and the results were expressed as mean ± standard deviations. Plotting was performed using Origin 2021, and significance analysis was performed using IBM SPSS 20, with a significance level of *p* < 0.05.

## 3 Results and discussions

### 3.1 Effect of EAA on the yield of pigment and citrinin

**Figure 2:**
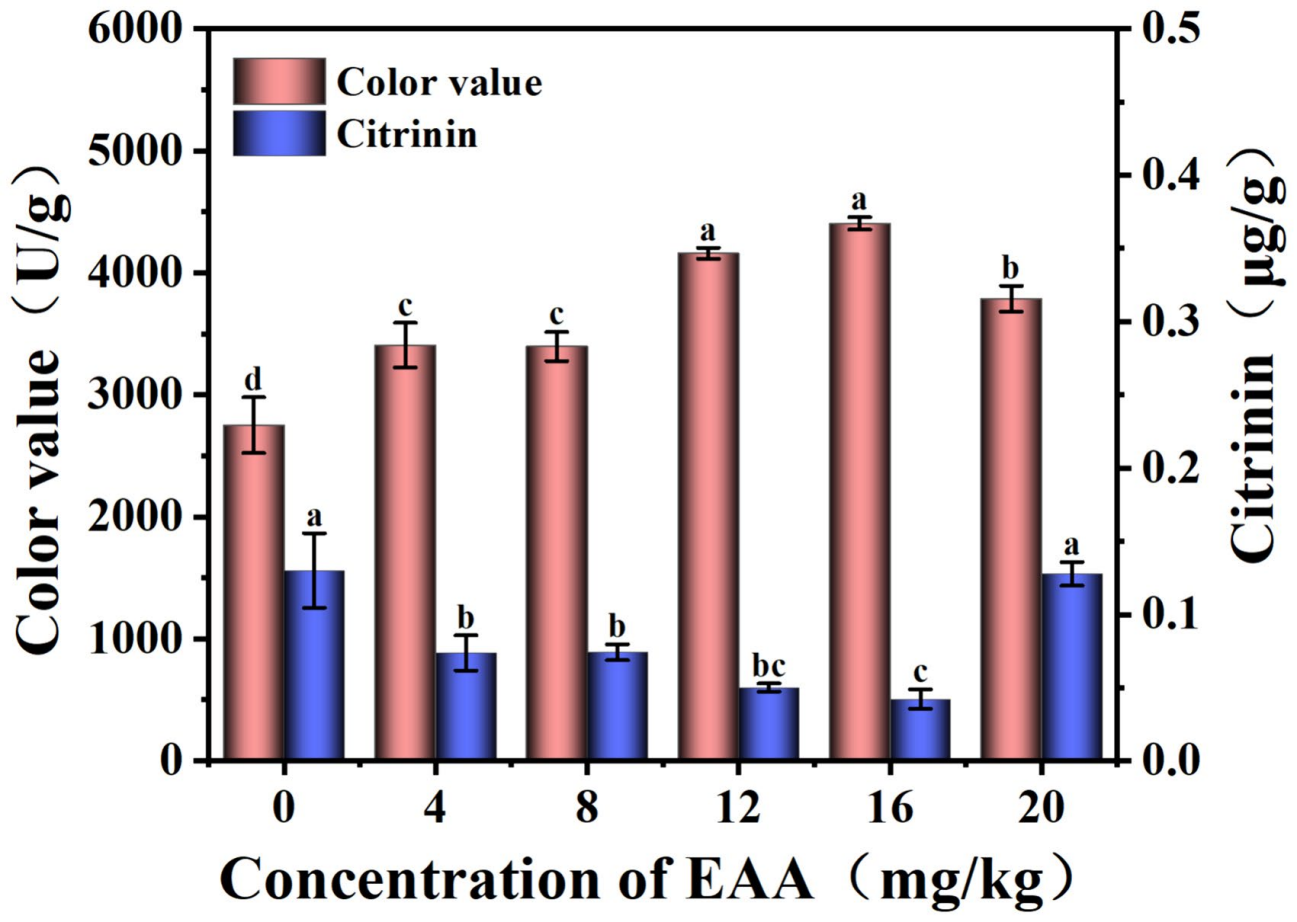
Effect of EAA on the yield of pigment and citrinin.

To explore the effect of EAA on the pigment and citrinin of red yeast rice, different concentrations of EAA were introduced. As shown in Figure 2, the color value presented a significant difference and exhibited the highest yield of 4,403 U/g at the concentration of 16 mg/kg, which increased by 60.1% compared to the blank group. As the EAA concentration increased, the citrinin levels initially decreased and then increased. The citrinin content was lowest at 0.042 μg/g when the EAA concentration of 16 mg/kg, representing a 67.6% reduction compared to the blank group. Therefore, 16 mg/kg was chosen as the optimal concentration for regulating red yeast rice fermentation.

### 3.2 Dynamic changes in physicochemical indices of red yeast rice

Based on the optimization of EAA concentration, the dynamic changes in pigment, citrinin, antioxidant activity, and functional enzyme activity of red years rice, with or without EAA addition, were researched.

**Figure 3:**
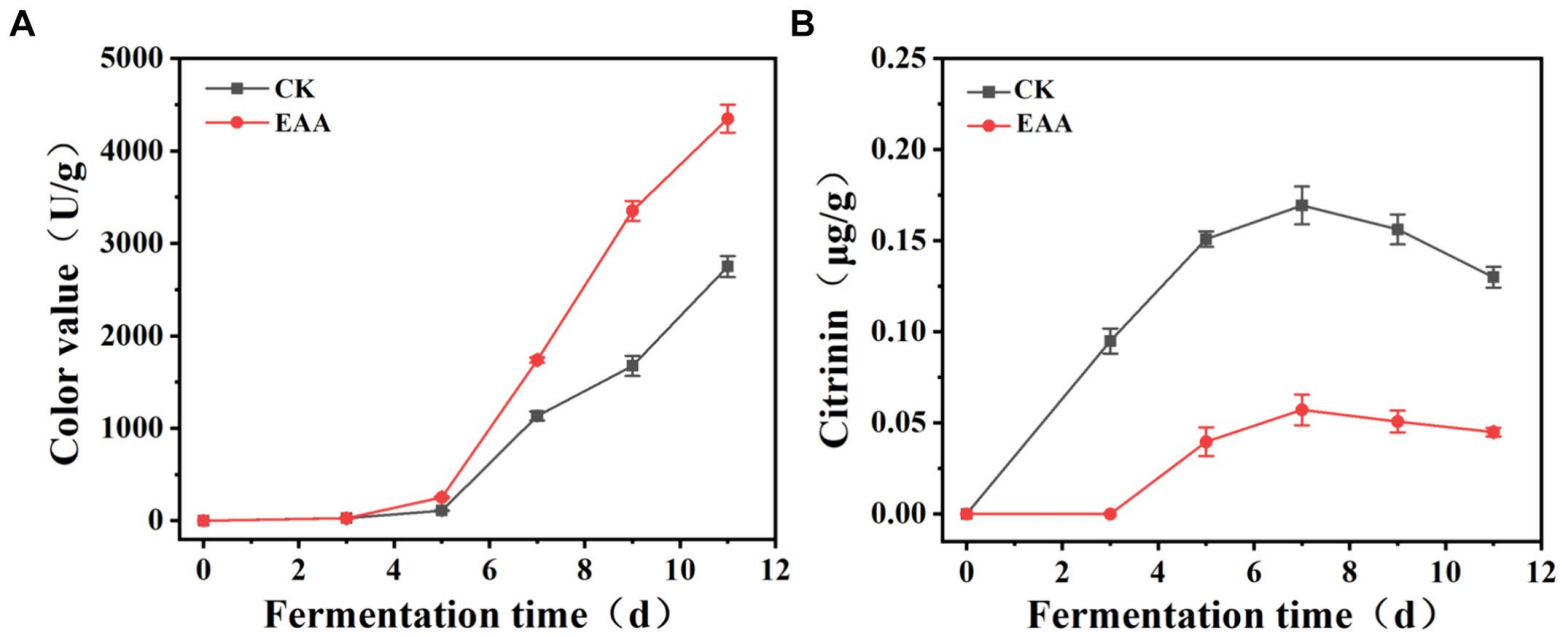
Dynamic changes in color value **(A)** and citrinin **(B)** during the fermentation of red yeast rice. CK: the blank group without the addition of EAA. EAA: the experience group with the addition of EAA.

During the fermentation of red yeast rice, the color value in both two groups gradually increased and exceeded 1,000 U/g on the 7th day (Figure 3A). At the middle and late stages of fermentation, the color value of the EAA group was significantly higher than that of the blank group and reached 4,350 U/g on the day 11, demonstrating the positive effect of the EAA on improving the pigment yield. Citrinin, a harmful secondary metabolite produced by *Monascus*, is synthesized via the polyketide pathway (He and Cox, 2016). As shown in Figure 3B, at the early stage of fermentation, citrinin increased in both groups, and the slope of the blank group was significantly higher than that of the EAA group. The citrinin level reached a maximum of 0.16 and 0.06 μg/g, respectively, in the blank and EAA groups on the 7th day. It can be speculated that EAA may affect the synthesis of citrinin by influencing the growth of *Monascus* at the early and middle stages of fermentation. At the late stage of fermentation, the content of citrinin decreased in both two groups with the content of 0.13 and 0.044 μg/g, respectively, in the blank and EAA groups on day 11, which might be related to the degradation of citrinin during the fermentation (Bazin et al., 2013).

**Figure 4:**
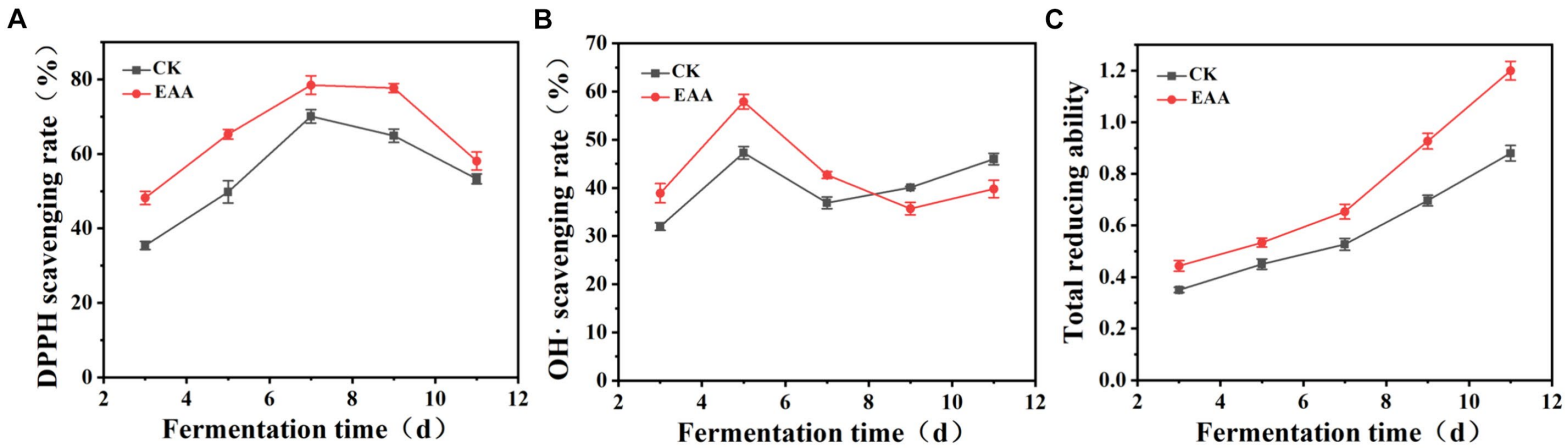
Dynamic changes in antioxidant activity during the fermentation of red yeast rice. **(A)** DPPH. **(B)** OH•. **(C)** Total reducing ability.

DPPH is a colored free radical, which is stable at room temperature (Foti, 2015). As shown in Figure 4A, the scavenging ability of DPPH free radicals in two groups presented a trend of initially increasing and then decreasing. During fermentation, the scavenging ability of the EAA group was higher than that of the blank group, reaching a maximum of 78.5 and 70.1%, respectively, on the 7th day. Hydroxyl radical is a kind of reactive oxygen that can cause serious damage to organisms (Liu et al., 2022). As shown in Figure 4B, the hydroxyl radical scavenging rate of the EAA group was significantly higher than that of the blank group from the 3rd day to the 7th day, reaching a maximum of 57.9% on the 5th day. Conversely, at the late stage of fermentation, the hydroxyl radical scavenging rate of the blank group was relatively higher. In addition, the total reducing power increased during the whole fermentation process, which was higher in the EAA group (Figure 4C). The results indicated that EAA can enhance the antioxidant activity of red yeast rice. Owing to the high antioxidant activity, the excessive free radicals generated via intracellular metabolism can be scavenged, ensuring the normal growth, reproduction, and metabolism of cells (Park and Kim, 2011).

**Figure 5:**
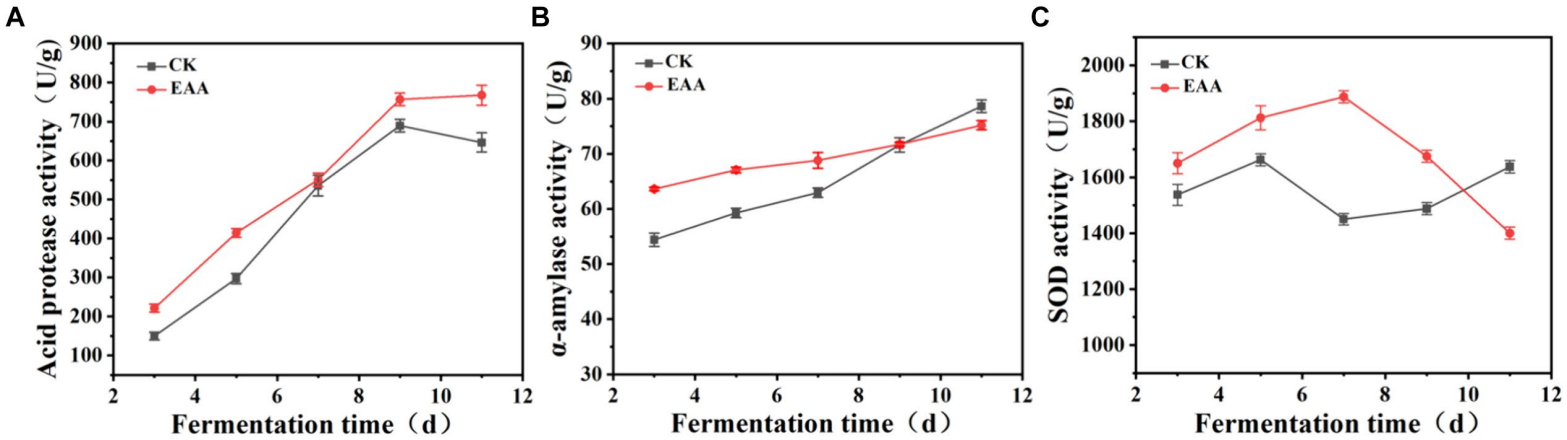
Dynamic changes in functional enzyme activity during the fermentation of red yeast rice. **(A)** Acid protease. **(B)** α-Amylase. **(C)** Superoxide dismutase.

Acid protease, a primary metabolite of *Monascus*, not only decomposes proteins into amino acids and peptides but also influences the formation of flavor precursors, thereby enhancing the flavor and quality of products (Rahayu et al., 2017). The dynamic changes in the acid protease during the fermentation were determined, and the results are shown in Figure 5A. Compared to the blank group, the addition of EAA enhanced the acid protease activity, which reached the maximum of 756.9 U/g on the 11th day. α-Amylase can translate starch to small molecules of dextrin, maltose, and glucose via hydrolyzing the α-1,4-glycosidic bond, providing nutrients for the growth of microorganisms (Long et al., 2020). Meanwhile, the interaction of α-amylase and acid protease can promote the formation of flavor substances (Zhu et al., 2022). As shown in Figure 5B, at the early stage of fermentation, the α-amylase activity of the EAA group was much higher than that of the blank group. From the 7th day, the increased speed of α-amylase activity was faster in the blank group. On day 11, the α-amylase activity reached the maximum of 78.6 and 75.2 U/g, respectively, in the blank and EAA groups. SOD plays a crucial role in the antioxidant defense of organisms, promoting the disproportionation reaction of superoxide anion into oxygen and hydrogen peroxide (Case, 2017). At the end of fermentation, the SOD activities were 1637.5 and 1,400 U/g, respectively, in the blank and EAA groups (Figure 5C).

The results indicated that EAA promoted the functional enzyme activity of red yeast rice, which is conducive to the decomposition of starch into small molecule substances, promoting the growth, reproduction, and metabolism of *Monascus*. The significant difference in SOD activity between the two groups at the early stage of fermentation indicated that EAA improved the ability of *Monascus* to oxidative stress.

### 3.3 Scan electron microscopy characterization of the red yeast rice

The above results demonstrated that EAA significantly improved the yield of the pigment, decreased the content of citrinin, and changed the antioxidant activity and functional enzyme activity of red yeast rice. Thus, it was speculated that EAA would also affect the microstructure of red yeast rice.

**Figure 6:**
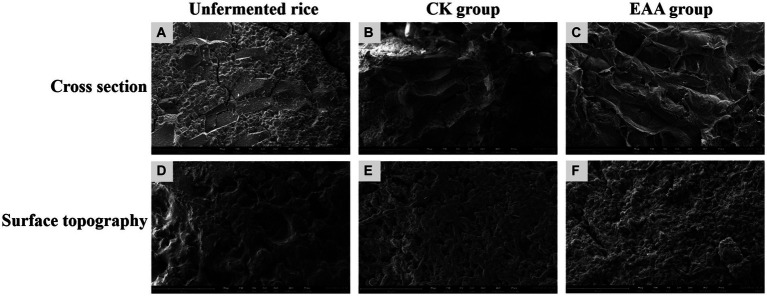
SEM images of the unfermented rice **(A,D)**, red yeast rice without EAA addition **(B,E)**, and red yeast rice with EAA addition **(C,F)**.

The microstructure of the truncated surface and the intact surface of the red yeast rice, with and without EAA addition, were observed using scanning electron microscopy. As shown in Figure 6, the unfermented rice presented a dense structure with fewer pores. Compared to the unfermented rice, the porous structure exhibited on the intact surface of red yeast rice fermented by strain M7-5 may be caused by the growth of *Monascus*. When EAA was introduced, the intact surface became loose and porous. The results showed that EAA significantly changed the microstructure of red yeast rice, which may be owing to amylase and protease produced by *Monascus* decomposing starch into small molecule substances, making the structure of the red yeast rice looser, which is conducive to the oxygen circulation and the entry of the mycelium (Lu et al., 2013).

### 3.4 Overview of RNA sequencing

In the present study, six samples were constructed for the two experimental groups, and six cDNA libraries (CK1, CK2, CK3, EAA1, EAA2, and EAA3) were subjected to high-throughput sequencing. After quality control and data filtering, a total of 47,768,980, 45,789,920, 45,705,526, 46,181,286, 45,759,274, and 41,426,612 clean reads were obtained (Supplementary Table S2). The GC contents ranged from 52.51 to 53.05%. The Q30 and Q20 values were greater than 93% and 97%.

The HISAT2 software was applied to quickly and accurately compare the clean reads to the reference genome to obtain the localization information of the reads. As shown in Table 1, the total maps were all above 96%, indicating a high alignment rate. The distribution of gene expression in the CK and EAA groups was stable and concentrated in the same region (Supplementary Figure S2).

**Table 1:** Comparative analysis of sequencing data quality.

Sample	Total reads	Total map	Unique map	Multi map
CK1	47,768,980	46,539,759 (97.43%)	45,592,474 (95.44%)	947,285 (1.98%)
CK2	45,789,920	44,437,396 (97.05%)	43,443,556 (94.88%)	993,840 (2.17%)
CK3	45,705,526	44,178,779 (96.66%)	43,290,313 (94.72%)	888,466 (1.94%)
EAA1	46,181,286	44,675,533 (96.74%)	43,773,690 (94.79%)	901,843 (1.95%)
EAA2	45,759,274	44,405,745 (97.04%)	43,497,128 (95.06%)	908,617 (1.99%)
EAA3	41,426,612	40,521,021 (97.81%)	39,583,734 (95.55%)	937,287 (2.26%)

**Figure 7:**
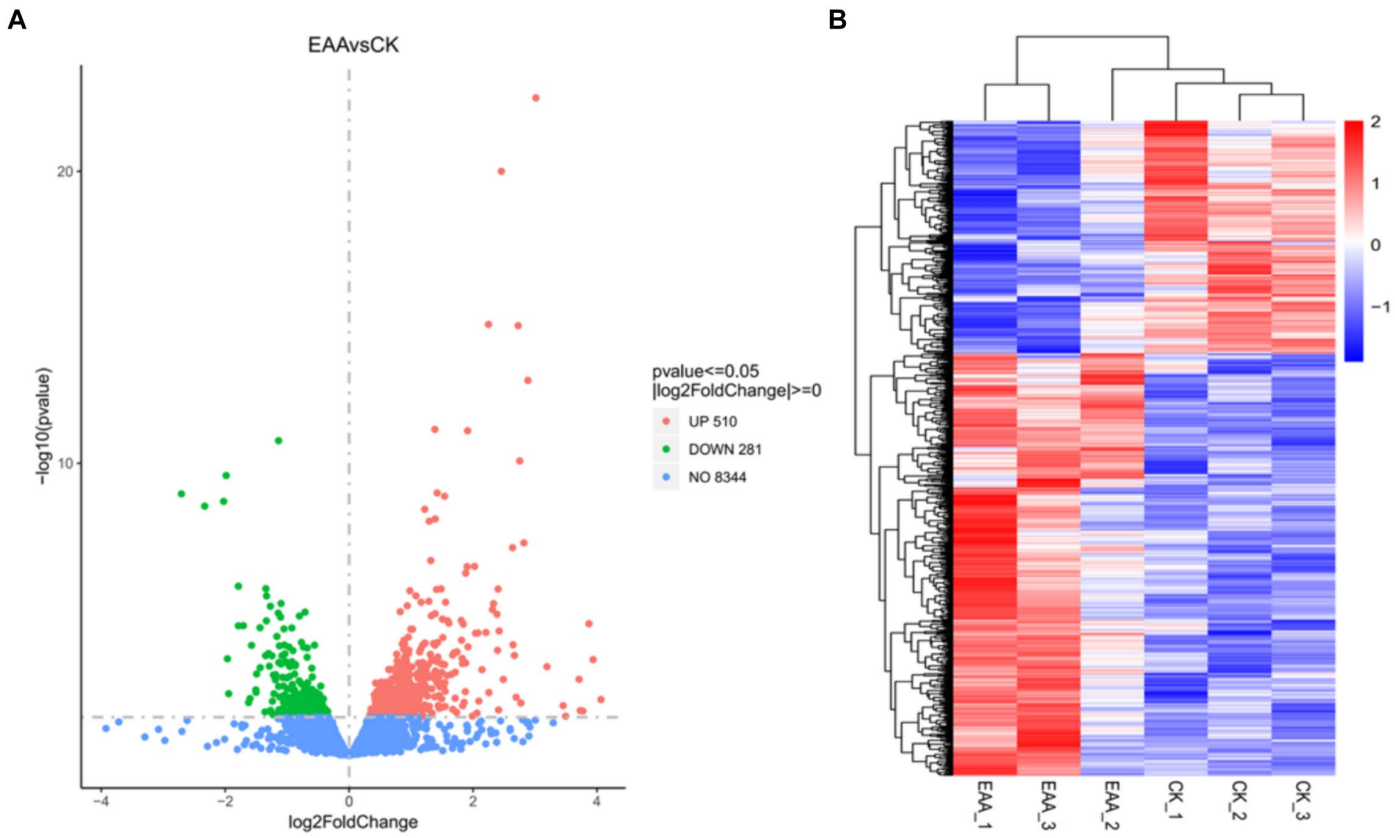
Analysis of differentially expressed genes. **(A)** Volcano plot. **(B)** Heatmap.

Fold change was utilized to assess the difference in gene expression among samples. The conditions for differential gene screening were: *p*-value <0.05 and |log_2_FoldChange| > 0. As the volcano plot of differentially expressed genes in the two groups shown in Figure 7A, a total of 791 differentially expressed genes were found in the EAA group vs. the CK group, among which 510 differentially expressed genes were upregulated and 281 differentially expressed genes were downregulated. A cluster analysis heatmap of the different genes intuitively revealed the difference of differentially expressed genes between the two groups (Figure 7B).

### 3.5 GO and KEGG functional annotation analysis

**Figure 8:**
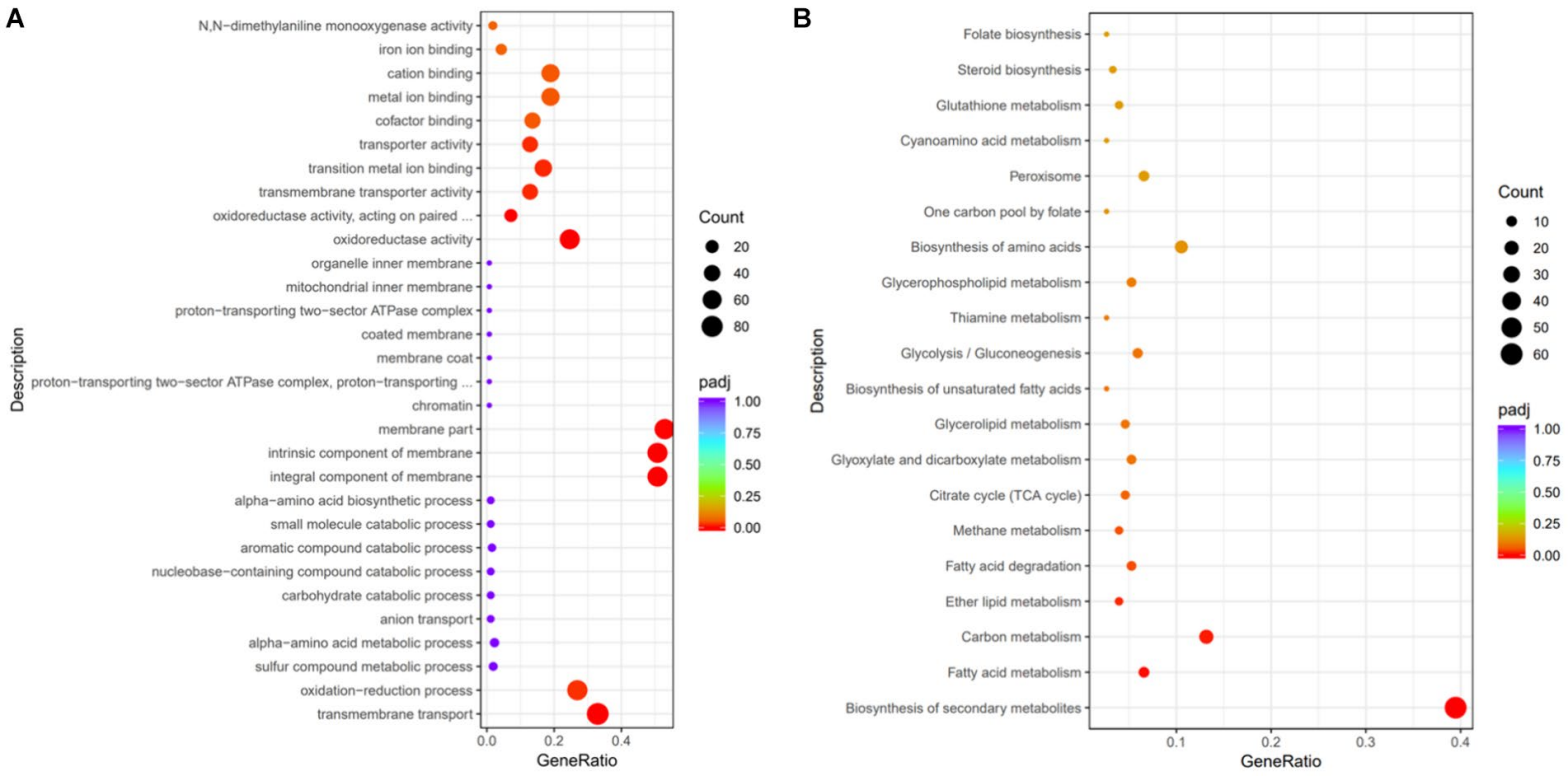
Functional annotation analysis of the differentially expressed genes. **(A)** GO functional analysis. **(B)** KEGG pathway analysis.

GO is a comprehensive database that describes gene functions, categorizing them into biological process (BP), cellular component (CC), and molecular function (MF). In the GO database, a total of 723 different entries between the two groups were detected, among which 352 terms belonged to MF, 213 terms belonged to CC, and 158 terms belonged to BP. The significantly enriched GO entries are shown in Figure 8A, where the bubble size represents the number of differential genes, and the bubble color represents the padj value.

The significantly enriched GO terms are shown in Table 2. Eight GO terms were significantly enriched and belonged to MF. GO:0016021 (overall membrane composition correlation), GO:0031224 (intrinsic membrane composition correlation), and GO:0044425 (partial membrane composition correlation) were significantly enriched and belonged to CC. GO:0055085 (transmembrane transport-related) and GO:0055114 (redox process-related) were significantly enriched and belonged to BP. The most significantly enriched GO:0016491 and GO:0016705 were both related to oxidoreductase activity, presenting 46 and 18 upregulated genes, respectively, which was consistent with the results that EAA increased functional enzyme activity of red yeast rice (Figure 5). GO:0016021, GO:0031224, and GO:0055085 were all related to membrane composition and transmembrane transport, and 56, 56, and 71 genes were upregulated, respectively, indicating that EAA changed the composition of the cell membrane, which led to the consequent change in membrane permeability, strengthen the transmembrane transport ability of pigment, and weakening the intracellular product inhibition.

**Table 2:** Significantly enriched GO terms.

Number	GO ID	Term type	Description	Ratio in pop	P. adjust
87	GO:0055085	BP	transmembrane transport	453/2457	0.0001
71	GO:0055114	BP	oxidation–reduction process	448/2457	0.016
70	GO:0016021	CC	integral component of membrane	402/1248	0.0001
70	GO:0031224	CC	intrinsic component of membrane	402/1248	0.0001
73	GO:0044425	CC	membrane part	456/1248	0.0003
69	GO:0016491	MF	oxidoreductase activity	442/3292	0.0001
20	GO:0016705	MF	oxidoreductase activity, acting on paired donors, with incorporation or reduction of molecular oxygen	63/3292	0.0001
36	GO:0022857	MF	transmembrane transporter activity	237/3292	0.011
47	GO:0046914	MF	transition metal ion binding	339/3292	0.011
36	GO:0005215	MF	transporter activity	242/3292	0.012
38	GO:0048037	MF	cofactor binding	283/3292	0.048
53	GO:0046872	MF	metal ion binding	433/3292	0.048
53	GO:0043169	MF	cation binding	434/3292	0.049

To identify the substances’ metabolism and their related signal pathways during red yeast rice fermentation, a metabolic pathway analysis was performed using the KEGG database (Figure 8B). A total of 79 metabolic pathways were annotated between the two groups. A total of 60 genes were found in the biosynthesis of secondary metabolites, among which 21 were upregulated, suggesting that EAA significantly affected the secondary metabolism network of *Monascus*. A total of 20 entries were found in carbon metabolism, among which 6 were upregulated. Additionally, the biosynthesis of amino acids contained 16 genes, among which 11 were downregulated. Fatty acid metabolism involved 10 genes, among which 7 were downregulated. These results indicated that carbon metabolism, amino acid metabolism, and fatty acid metabolism were closely related to the biosynthesis of pigment and citrinin. In carbon metabolism, the gene-MAP00_001419 (encoding pyruvate kinase, which is the rate-limiting enzyme of glycolysis) and gene-MAP00_003655 (encoding fructose-1,6-bisphosphatase) related to the glycolytic pathway, were upregulated. In addition, two key rate-limiting enzymes, gene-MAP00_006738 (encoding citrate synthase) and gene-MAP00_001053 (encoding isocitrate dehydrogenase) in the TCA cycle were both upregulated, suggesting that EAA enhanced the sugar metabolism capacity, which can provide sufficient energy for the production of secondary metabolites, as well as provide more precursors for the synthesis of pigment, benefiting for the growth of M7-5 and production of pigment (Huang et al., 2021).

In the EAA group, the expression of several key genes in the amino acid degradation pathway was significantly upregulated, including the gene MAP00_006064 (log_2_FC 2.88, encoding 5-carboxylate reductase-like protein), MAP00_006738 (log_2_FC 1.14, encoding 2-methylcitrate synthetase) and MAP00_001587 (log_2_FC 1.22, encoding 3-isopropyl malate dehydratase). At the same time, genes involved in the amino acid biosynthesis pathway were downregulated, including the gene MAP00_005180 and MAP00_000342, which encode serine hydroxymethyltransferase and S-adenosine methionine synthetase, respectively. The different expressions of these genes may indicate changes in amino acid metabolism in cells. Since amino acids are precursors of citrinin and pigment biosynthesis, the regulation of their metabolism directly affects the production of secondary metabolites, further contributing to the high pigment yield and low citrinin yield. In addition, genes related to the fatty acid degradation pathway were upregulated in the EAA group, including MAP00_007588 (log_2_FC1.03, encoding ethanol dehydrogenase) and MAP00_006805 (log_2_FC0.893, encoding dienoyl-CoA isomerase). The gene expression in the fatty acid synthesis pathway was downregulated, such as the gene MAP00_008691 (log_2_FC1.35, encoding the β-fatty acid synthetase subunit) and MAP00_004941 (log_2_FC1.44, encoding oleate hydroxylase). The increase in fatty acid degradation may provide more energy and intermediate metabolites for pigment, while the decrease in fatty acid synthesis may reduce the substrate supply for citrinin.

### 3.6 Analysis of the pigment and citrinin-related metabolic pathways

**Figure 9:**
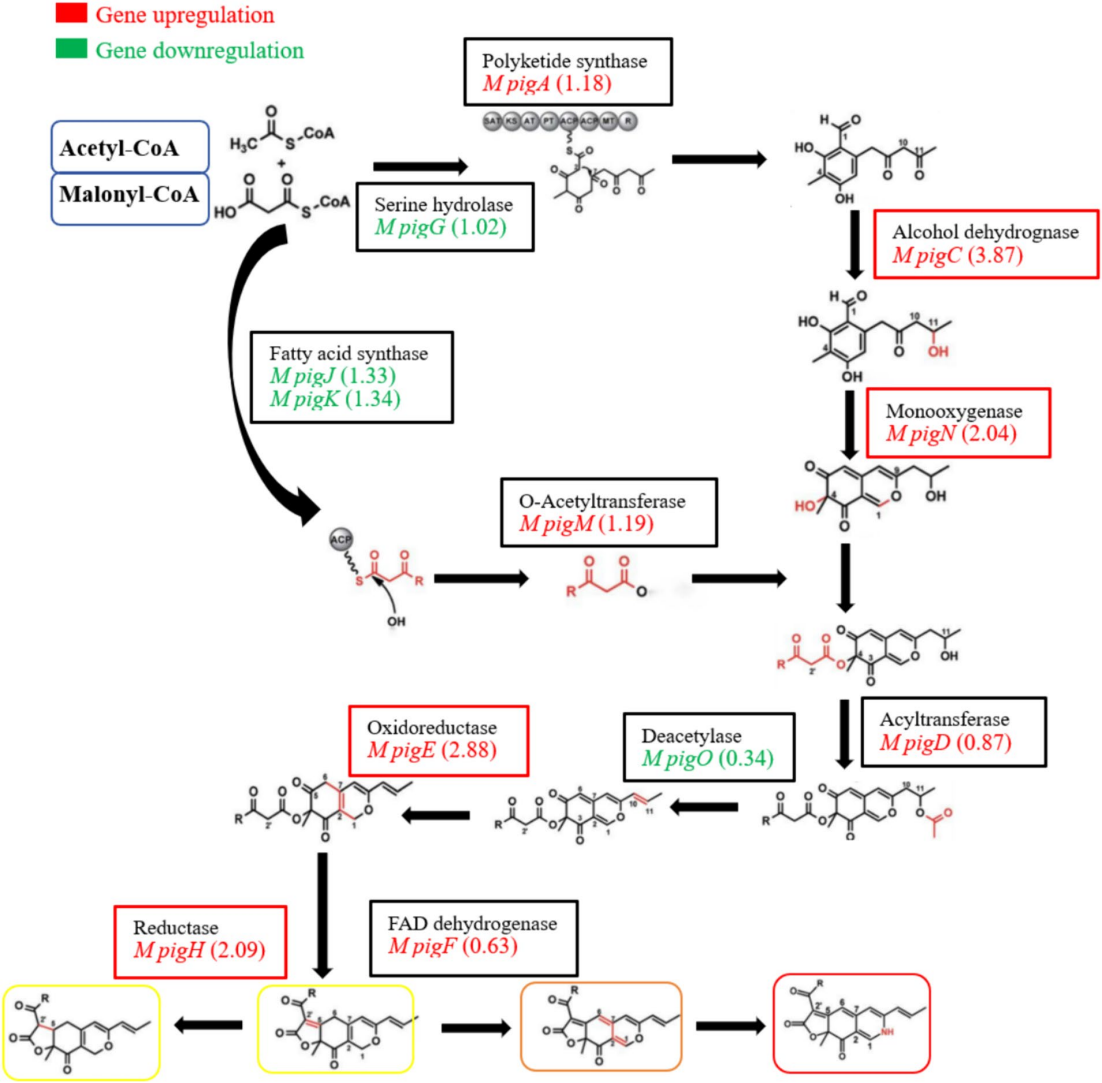
Differential expression analysis of genes related to the synthesis of pigment in red yeast rice.

Among the biosynthesis of secondary metabolites of red yeast rice, pigment has attracted much attention. It has been reported that the synthesis of pigment mainly depends on the PKS gene cluster, of which 12 key enzymes may be involved in the biosynthesis of pigments in strain M7-5 (Chen W, et al., 2017; Chen et al., 2019). As shown in Figure 9, compared to the blank group, EAA enhanced the transcript levels of most of the genes related to the synthesis of pigment. Among these, the expression level of four key enzymes, ethanol dehydrogenase (*M pigC*), oxidoreductase (*M pigE*), reductase (*M pigH*), and monooxygenase (*M pigN*), significantly increased by 3.87, 2.88, 2.09, and 2.04 log_2_FC, respectively. It was suggested that the increase in pigment may be related to the over-expression of *M pigC*, *M pigE*, *M pigH,* and *M pigN*. According to Liang’s report, after 8 days of incubation for *M. purpureus YY-1*, pigment content increased rapidly. Genes expression levels of PKS, short-chain alcohol dehydrogenase, 3-o-acetyltransferase, reductase-like proteins, and fatty acid synthase were high, which coincided with our research (Liang et al., 2018).

In addition, in strain M7-5, EAA downregulated the expression of serine hydrolase (*M pigG*), fatty acid synthase (*M pigJ* and *M pigK*), and deacetylase (*M pigO*). Yin’s research showed that histidine and methionine exhibited significantly different influences on the yield of pigment. However, the expression of reductase-like proteins, fatty acid synthetases, and deacetylases was downregulated in the transcriptional profiles of the genes in two groups (Yin et al., 2022). It can be hypothesized that serine hydrolase (*M pigG*), fatty acid synthase (*M pigJ* and *M pigK*), and deacetylase (*M pigO*) have limited influence on pigment synthesis in strain M7-5.

The *Monascus* pigment PKS gene cluster can be controlled by cluster-specific regulatory transcription factors, which play key roles in controlling gene expression (Liu J, et al., 2021). It has been reported that the transcriptional expression levels of the two regulatory transcription factors, *M pigB* and *M pigI,* in the PKS gene cluster were upregulated in *M. purpureus* RP2 strains with high pigment production. The same results were also obtained in *M. purpureus* M9 strain (Chen D, et al., 2017). In strain M7-5, the addition of EAA significantly upregulated the expression of two transcriptional regulators, *M pigB* (2.67) and *M pigI* (2.64), which may explain the higher pigment production in the EAA group.

**Figure 10:**
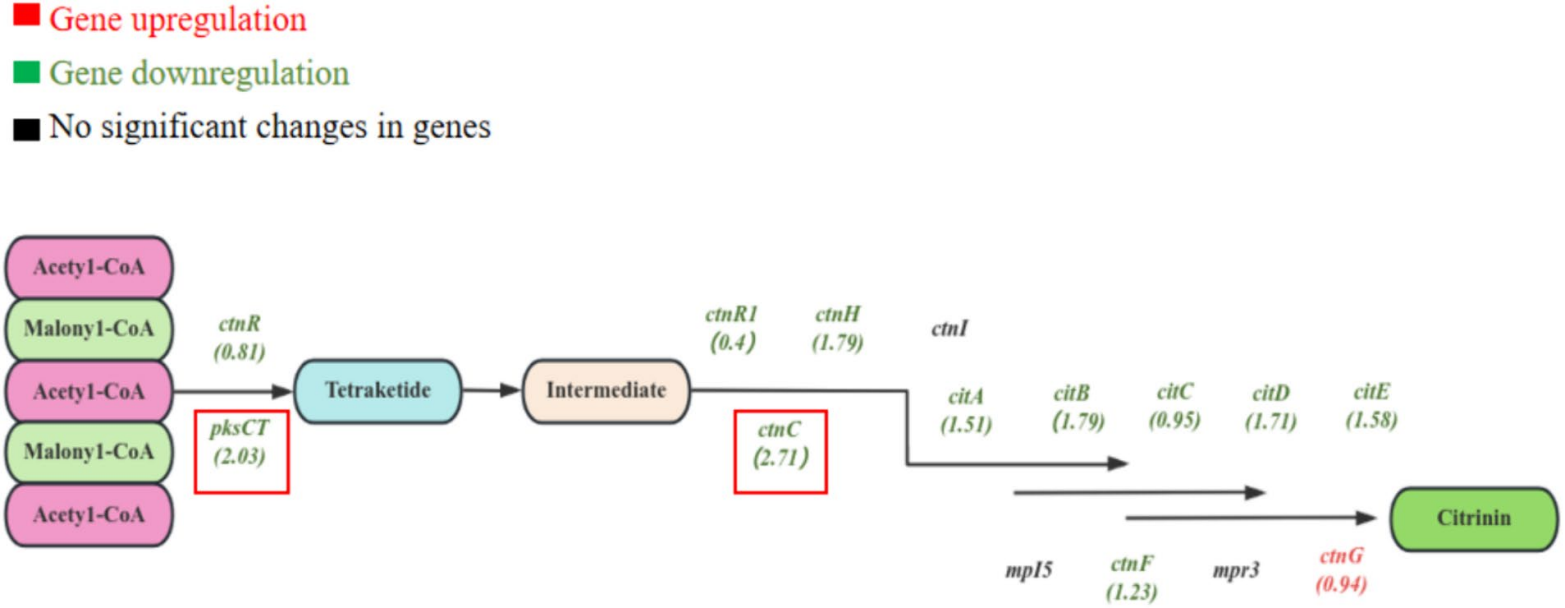
Differential expression analysis of genes related to the synthesis of citrinin in red yeast rice.

The genes related to citrinin biosynthesis were also explored. As the pathway of citrinin biosynthesis shown in Figure 10, most of the citrinin biosynthesis genes were differentially expressed (Huang Y, et al., 2023). Compared to the blank group, 11 genes related to citrinin biosynthesis were downregulated in the EAA group, among which the most significant ones were *ctnC* (2.71) and *pksCT* (2.03). Research has shown that *pksCT*, *ctnA* (transcriptional regulatory protein), *ctnB* (oxidoreductase), and *ctnE* (dehydrogenase) are positively regulating citrinin synthesis (He and Cox, 2016; Ning et al., 2017; Wan et al., 2017). Thus, the low expression of *pksCT* and *ctnC* may be the most important reason for the reduction of citrinin in the EAA group.

In addition, the downregulated log_2_FC values of *citA*, *citB*, *citD*, *citE*, and *ctnH* genes were all higher than 1.5, which was similar to the results of Chen (Liang et al., 2017). The deletion of the *citA* gene led to a significant decrease in the production of citrinin but did not affect the pigment (He and Cox, 2016). The *ctnH* gene may be involved in the post-modification process of citrinin biosynthesis (Ouyang et al., 2021). The *ctnF* gene was found to be associated with the accumulation of acetyl coenzyme A and malonyl coenzyme A, both of which are precursors of citrinin biosynthesis. Therefore, the decrease in the transcript level of *ctnF* may affect the synthesis of citrinin (Li et al., 2020). The *ctnR* was the transcriptional activator of citrinin biosynthesis, and its transcriptional level expression showed downregulation. In addition, *ctnI*, *mpI5*, and *mpr3* genes showed no significant changes. The above results suggest that EAA may control the synthesis of citrinin by affecting the transcription of the citrinin biosynthesis gene cluster in *Monascus*.

### 3.7 RT-qPCR validation of key genes

Eight upregulated genes (*M pigC*, *M pigE*, *M pigH*, *M pigN*, *M pigM*, *M pigA*, *ctnG*, and *M pigD*) and eight downregulated genes (*ctnF*, *M pigJ*, *M pigK*, *citA*, *citE*, *ctnH*, *citB,* and *pksCT*) were selected from the transcriptome data for RT-qPCR analysis to verify the accuracy of the transcriptome analysis. The information and differential expression level of the selected genes are shown in Supplementary Table S3. As shown in Supplementary Figure S3, the RT-qPCR data were consistent with the transcriptome results, indicating that the transcriptome data were reliable.

## 4 Conclusion

The results showed that EAA increased the pigment by 58.2% and decreased citrinin by 65.4%. EAA also induced an increase in acid protease activity, DPPH free radicals, total reducing power, a decrease in hydroxyl free radicals, α-amylase activity, and SOD activity. The microstructure of red yeast rice was significantly changed by the addition of EAA, which might be beneficial for the fermentation of *Monascus.* Transcriptomics was applied to reveal the effect of EAA on pigment and citrinin metabolic mechanisms. A total of 791 differentially expressed genes were identified, among which 510 genes were upregulated and 281 genes were downregulated. Carbon metabolism, amino acid metabolism, and fatty acid metabolism were the main metabolic pathways, which are closely related to the biosynthesis of pigment and citrinin. The pigment and citrinin metabolic pathways were also analyzed. *M pigC*, *M pigE*, *M pigH,* and *M pigN* of the PKS gene cluster may be the key genes for the biosynthesis of pigment, and *pksCT* and *ctnC* may be related to the biosynthesis of citrinin. The study demonstrated that EAA posed positive impacts on the increase of pigment and reduction of citrinin during the fermentation of red yeast rice, providing valuable experience for the in-depth research of pigment and citrinin metabolic mechanisms as well as product quality improvement. In addition, in future studies, the molecular mechanism of synthesis of pigment and citrinin in *Monascus purpureus* M7-5 can be further elucidated by knockout, mutation, or allogeneic expression of key genes.

## Data Availability

The original contributions presented in the study are included in the article/Supplementary material, further inquiries can be directed to the corresponding author.

## Footnotes

^1^ http://www.geneontology.org/

^2^ http://www.genome.jp/kegg/
