# Supplementary material for: Study on the effect of ascorbic acid on the biosynthesis of pigment and citrinin in red yeast rice based on comparative transcriptomics
Source: Front Microbiol. 2024 Sep 10;15:1460690. doi: 10.3389/fmicb.2024.1460690 (PMC11419985; doi:10.3389/fmicb.2024.1460690)
Supplement: Supplementary file 1 [file Data_Sheet_1.docx]

**Study on the effect of** **ascorbic acid on the biosynthesis of pigment and citrinin in red yeast rice based on** **comparative transcriptomic**


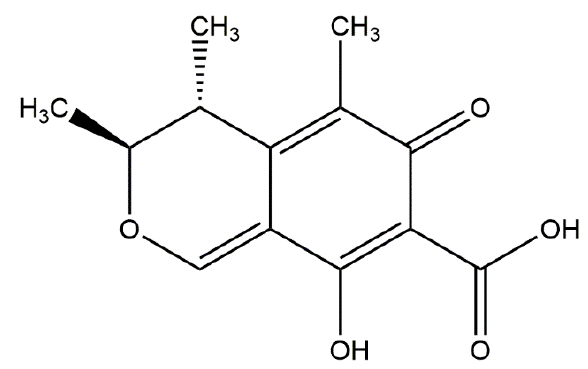


**Fig. S1**. The molecular formula of citrinin.


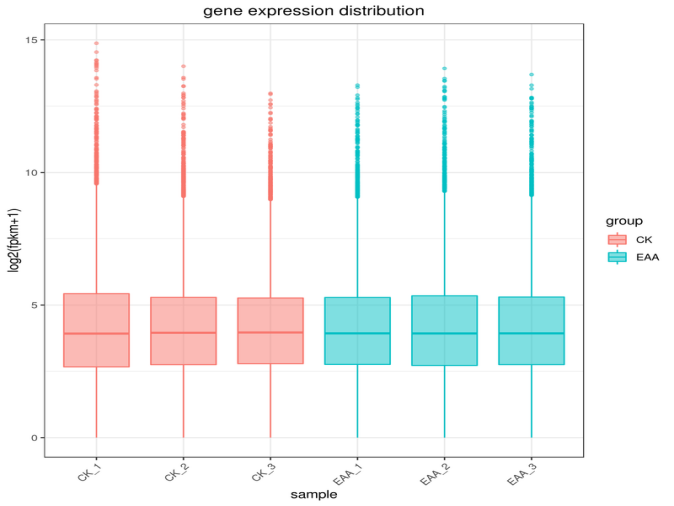


**Fig. S2**. Gene expression depth.


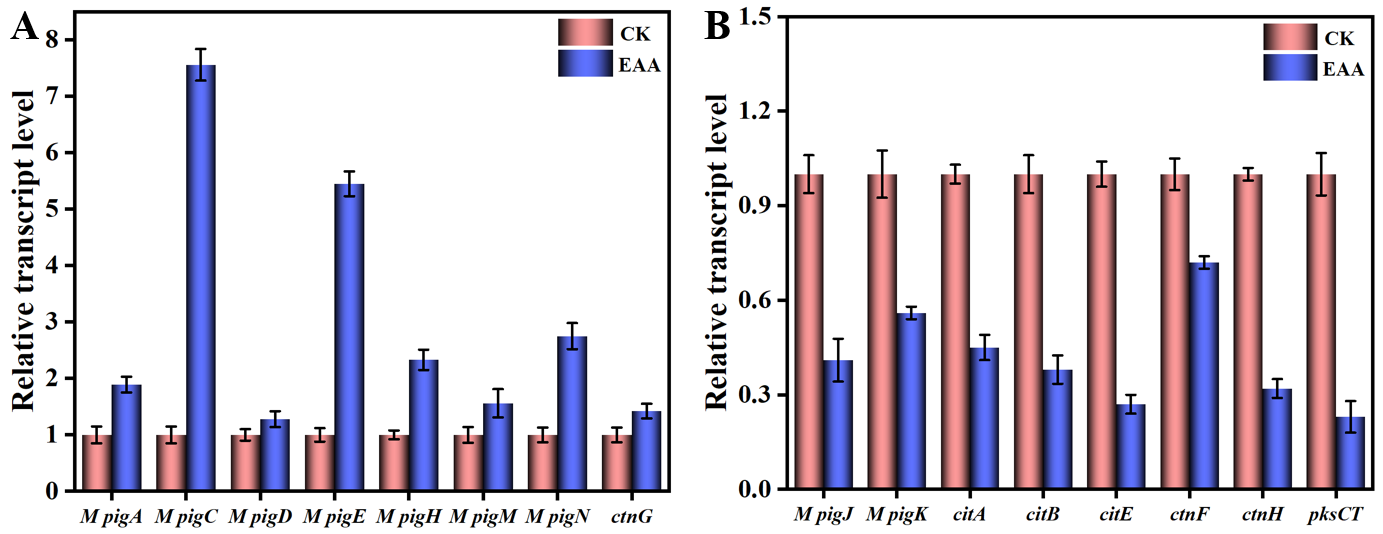


**Fig. S3**. RT-qPCR validation of key genes related to the biosynthesis of pigment and citrinin.

**Table S1**. Primers used for RT-qPCR.

| Primers name | Sequences (5′→3′) |
| --- | --- |
| M pigC F | GCTGATTTCGGGTGTTTC |
| M pigC R | GCTTGTTACTTTTGCTGTTC |
| M pigE F | AGCAAGTACACCAGGTACATACGC |
| M pigE R | TGGTTACCGTTGCTCCTTCGAAT |
| M pigH F | CTTCCCGATGCCGTTGTGAT |
| M pigH R | CGTCTCGTGGATCATCTCGT |
| M pigN F | GTCACGAAAGAAGAGCCACAGCT |
| M pigN R | TCCAATTCCGGCTCCGATTTCG |
| M pigM F | ATCCTTCAAACCGCTCAACCG |
| M pigM R | CGGACACTTTGTACAGTTTCGCAA |
| M pigA F | CCACAGTCAATCCAGCTCTT |
| M pigA R | GCAGCATCTACCCAGCTATATT |
| ctnG F | TTACAAAGTTCGTCGGGACAGT |
| ctnG R | GGAGGAGGTTGGGAGGTGTT |
| M pigD F | CGAGTCAAGGATGTGACGGC |
| M pigD R | GCTCACAGGAAATGCAGGCT |
| ctnF F | CCTCGCTGTCCTCGAAATA |
| ctnF R | CTGCTGGCACCCCTGTAG |
| M pigJ F | ATGGATCGCCCGATCTTGTC |
| M pigJ R | CTTTGTCGAGTCCGCTGGAT |
| M pigK F | CCTCCAGGGATTACAACCCG |
| M pigK R | ATTCAATGCCAGGTGCTCCA |
| citA F | GTGTCATCGACCACCTCTAAAT |
| citA R | CGGTATGTTCCAGGTTGAGATAG |
| citE F | CAAGGCAAAGTTGGTGGATTC |
| citE R | TCCCAGTTGGCACTCAA ATAG |
| ctnH F | GGACATTGCGAGTGTTAGAGCC |
| ctnH R | GGCGTTCACGGAAAGTTGGT |
| citB F | GAGCCATCACGCTTCTCTT |
| citB R | CGTCTTTGTTCGGAGGAACT |
| pksCT F | GTCCCATTGACAGACATCGC |
| pksCT R | TGGCACCAGTAACAAGCACA |
| GAPDH-F | CGATGCCCAGTAATTATTAGGAC |
| GAPDH-R | CTGTAGCGCCTGACCTCGTA |

**Table S2**. Quality statistics of sequencing data.

| Samples | Raw reads | Clean reads | Error rate (%) | Q20 (%) | Q30 (%) | GC (%) |
| --- | --- | --- | --- | --- | --- | --- |
| CK1 | 48207366 | 47768980 | 0.03 | 97.68 | 93.76 | 53.05 |
| CK2 | 46399130 | 45789920 | 0.03 | 97.77 | 93.95 | 52.82 |
| CK3 | 46275708 | 45705526 | 0.03 | 97.40 | 93.18 | 52.79 |
| EAA1 | 46648002 | 46181286 | 0.03 | 97.49 | 93.33 | 52.51 |
| EAA2 | 46376374 | 45759274 | 0.03 | 97.67 | 93.74 | 52.95 |
| EAA3 | 41763346 | 41426612 | 0.03 | 97.76 | 93.84 | 52.83 |

**Table S3**. Validation genes of transcriptome data and their differential expression levels.

| Gene_ID | Name | CK | T | log_2_FoldChange | Regulation |
| --- | --- | --- | --- | --- | --- |
| gene-MAP00_005737 | *M pigC* | 332.586 | 4863.603 | 3.870 | Up |
| gene-MAP00_006064 | *M pigE* | 30.351 | 219.721 | 2.885 | Up |
| gene-MAP00_005414 | *M pigH* | 2.375 | 10.247 | 2.093 | Up |
| gene-MAP00_005116 | *M pigN* | 221.293 | 910.596 | 2.040 | Up |
| gene-MAP00_006481 | *M pigM* | 4658.382 | 10598.820 | 1.186 | Up |
| gene-MAP00_001488 | *M pigA* | 138.458 | 312.591 | 1.176 | Up |
| gene-MAP00_008577 | *ctnG* | 38.094 | 73.649 | 0.944 | Up |
| gene-MAP00_003970 | *M pigD* | 183.791 | 335.122 | 0.867 | Up |
| gene-MAP00_005182 | *ctnF* | 18749.260 | 8014.778 | -1.226 | Down |
| gene-MAP00_008690 | *M pigJ* | 55177.950 | 21897.160 | -1.333 | Down |
| gene-MAP00_008691 | *M pigK* | 15106.920 | 5952.814 | -1.344 | Down |
| gene-MAP00_006372 | *citA* | 9598.582 | 3381.756 | -1.505 | Down |
| gene-MAP00_006376 | *citE* | 8010.934 | 2689.704 | -1.575 | Down |
| gene-MAP00_006374 | *ctnH* | 17419.880 | 5053.193 | -1.785 | Down |
| gene-MAP00_006378 | *citB* | 8642.727 | 2505.203 | -1.787 | Down |
| gene-MAP00_006380 | *pksCT* | 9566.557 | 2350.403 | -2.025 | Down |
